# Commercial determinants of oral health in India: implications for research and policy- a perspective

**DOI:** 10.3389/froh.2026.1836443

**Published:** 2026-08-07

**Authors:** Lathadevi Chilgod, Rajeev B R, Malu Mohan, Aswathy Ram, Raveena Raveendran, Ramya Krishna B M, Upendra Bhojani

**Affiliations:** 1 Centre for Health Systems, Institute of Public Health Bengaluru, Bengaluru, Karnataka, India; 2 Centre for Commercial Determinants of Health, Institute of Public Health Bengaluru, Bengaluru, Karnataka, India

**Keywords:** commercial determinants of health, health policies, India, oral health, political economy

## Abstract

Oral diseases remain a major global public health challenge, with a disproportionate burden in low- and middle-income countries (LMICs) such as India. However, research and policy in oral health continue to focus predominantly on individual behaviours and clinical care, with limited attention to upstream determinants. This perspective piece applies the framework of commercial determinants of health to examine oral health in India, demonstrating how commercial actors shape disease risk and access to care. We examine three domains: (i) commodities related to oral health, including both harmful products and beneficial goods and services; (ii) structural conditions, such as economic liberalization, governance gaps, and societal norms that enable commercial influence; and (iii) market, business, and political strategies used by commercial actors to expand and sustain markets. Through interconnected pathways across these domains, commercial determinants contribute to differential exposure and access, reinforcing oral health inequities. We argue that advancing oral health equity in India requires us to expand beyond biomedical approaches towards integrating political economy and social science perspectives in research and policy. We also outline key research directions and methodological approaches to strengthen the evidence base on commercial determinants of oral health and inform more effective and equitable policy responses.

## 1 Introduction

Public health scholarship increasingly recognizes the complex relationship between commercial interests and health. The production and global marketing of unhealthy commodities, such as sugar-sweetened beverages, ultra-processed foods, tobacco, and alcohol, are major drivers of the non-communicable disease (NCD) burden (1). These “systems, practices, and pathways through which commercial actors influence health” are conceptualized as the Commercial Determinants of Health (2, 3).

Oral diseases affect an estimated 3.5 billion people globally, with a disproportionate burden in low-and middle-income settings (LMICs) (4). Yet oral healthcare systems remain predominantly curative, with limited emphasis on prevention (4). This narrow focus masks the upstream commercial forces shaping both disease risk and access to care. Although the FDI World Dental Federation recognizes commercial determinants as critical to oral health, research continues to focus largely on individual behaviours rather than the complex dynamics governing the production, pricing, and marketing of the risk factors (5).

Despite decades of research into risk factors, significant analytical gaps persist, particularly in South Asian contexts where corporate presence has intensified and expanded into emerging markets. For instance, substances such as betel quid, gutkha, and arecanut have been extensively studied for their health effects but are rarely analysed as commercial products embedded in specific production and distribution systems. The evidence base remains skewed toward high-income settings, reducing its relevance in LMICs (6). Another under-examined area is the pricing and marketing strategies used by commercial actors to target vulnerable populations.

These dynamics are particularly evident in India, where a rapidly expanding commercial ecosystem, characterized by the coexistence of formal corporate sectors and extensive informal markets (7, 8). Limited public spending in oral healthcare, weak regulatory oversight of the private dental sector, and affordability barriers exacerbate oral health inequities (7, 9). In this perspective, we advance a conceptual framework for understanding the Commercial Determinants of Oral Health in India, and outline key directions for future research on how these forces shape oral health inequities.

## 2 Conceptual framework for commercial determinants of oral health

**Figure 1:**
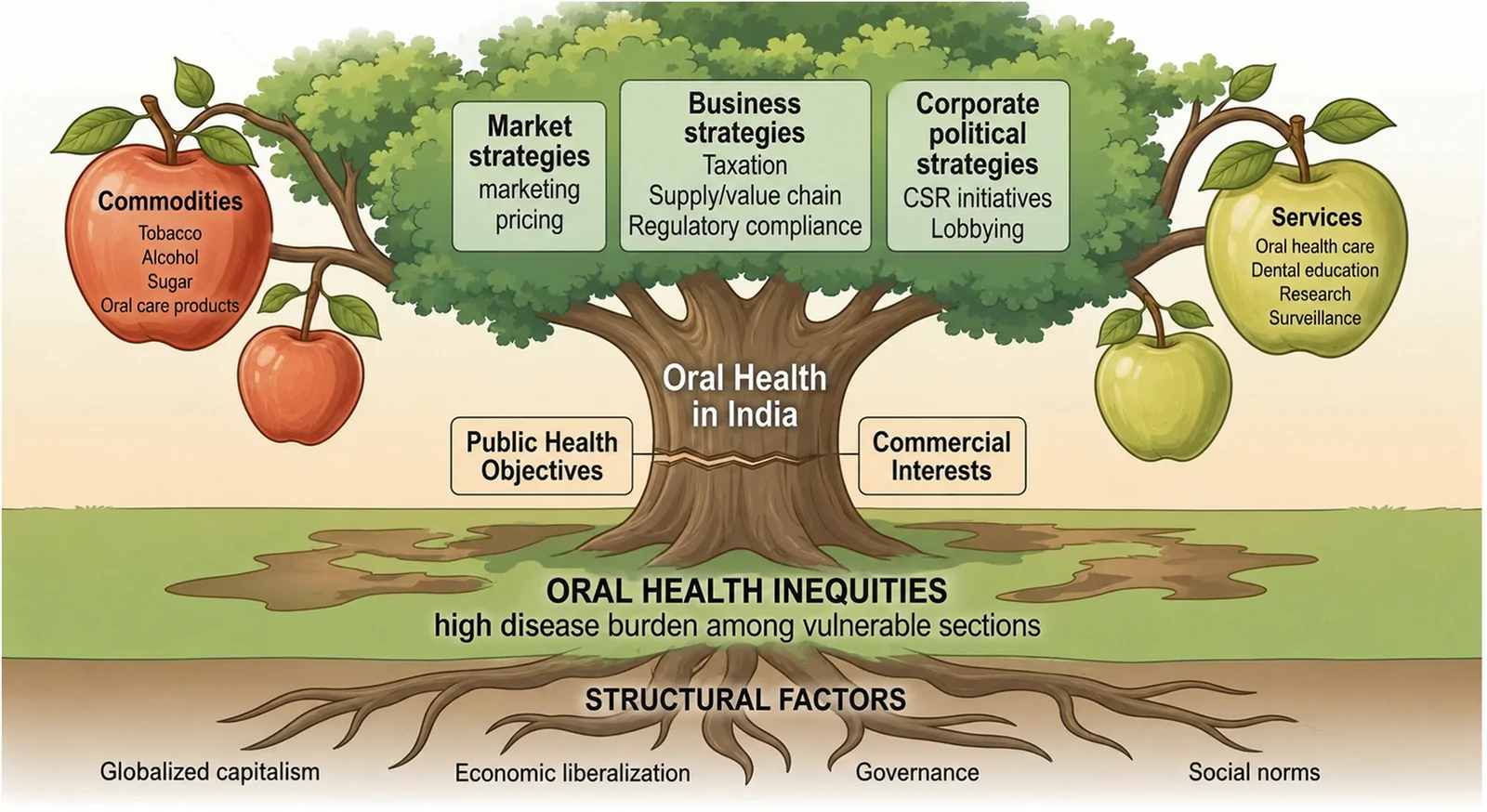
Conceptual framework depicting how commercial determinants shape oral health in India, informed by the commercial determinants of health framework proposed by Mialon M. (2020).

Drawing on existing scholarship, we examine three interrelated domains: (i) commodities related to oral health, (ii) structural conditions shaping commercial power, and (iii) market, business, and political strategies through which commercial actors influence health (Figure 1) (10, 11).

### 2.1 Commodities as determinants of oral health

A range of oral health-related commodities presents a tension between commercial and public health objectives (Figure 1). Tobacco and alcohol have long been recognized within the public health discourse, including oral health, though often without explicit attention to the commercial side. While the role of sugar as a primary risk factor for dental caries has been recognized for over a century, its framing as a commercial determinant has gained prominence more recently, particularly as a common risk factor for a range of non-communicable diseases (12, 13).

The commercial determination of oral health is not limited to harmful commodities like tobacco. Dentifrices and other oral care products, while offering preventive benefits, are marketed and priced in ways that shape access, perceptions, and consumption (14). Dental education and oral healthcare delivery themselves have increasingly been organized around market logic, contributing to the commodification of professional training and care provision (14, 15). Similar concerns regarding commercialization, oversupply of dental professionals, and inequitable access within market-oriented oral healthcare systems have been discussed in other Global South contexts, such as Chile (16). The growing dependence on commercially generated oral health data, in the context of weak public surveillance and research, represents another under-recognized commercial influence with implications for equity and governance (17).

### 2.2 Structural drivers of oral health

Over the past two decades, there has been a growing recognition of structural factors driving oral health risks and inequities (18). Drawing on Lee and colleagues’ conceptualization of the commercial determinants of health, we understand structural factors as the socio-political and economic conditions, governance and regulatory arrangements, and societal norms that shape how commercial power is produced, legitimized, and exerted, within oral health systems (19).

The macro-structural transformation of the country's economy since the early 1990s has fundamentally changed India's economic landscape from welfare-oriented goals toward a market-driven logic. This led to growing privatization across sectors such as healthcare and professional education, including dentistry, resulting in an urban concentration of services and widening geographic inequities (17). The drastic and poorly regulated growth of private dental colleges has saturated the job market with dental graduates and intensified competition within an already largely private oral healthcare system. Evidence from India suggests that this expansion has been accompanied by high levels of unemployment, financial vulnerability, and dependence on private-sector employment among young dentists. In this context, market-oriented models of practice may become increasingly normalized, contributing to the commodification of oral healthcare delivery (15, 20).

Persistent gaps in public policy and regulatory governance have also significantly contributed to the commercialization of oral health and healthcare in India. The absence of a national oral health policy and a clear vision for oral health have led to limited public financing, weak integration into primary care, and near-total lack of insurance coverage for dental services. In this policy vacuum, oral healthcare has been almost entirely ceded to market forces, resulting in private-sector dominance with implications for affordability and accessibility (21).

Regulatory weaknesses are also evident in the governance of harmful commodities, particularly in implementing bans on smokeless products such as gutkha, which have detrimental oral health impacts. Despite statutory restrictions, surrogate advertising, uneven enforcement of National Tobacco Control Policy (NTCP) regulations, and limited engagement with fiscal policies such as sugar taxation persist (22). The conflicts of interest within the policymaking processes, which allow the commercial actors to influence policies through lobbying and industry partnerships, continue to undermine the efforts to prioritize population oral health and equity (23).

Commercial influence is also sustained through the shaping of social norms that normalize consumption and individual responsibility for oral health. Marketing and advertising play a central role in this process by shaping the preferences, meanings, and aspirations of commodities and services related to health (2, 10). They also advance the idea that consumption itself is a normative marker of social status and mobility, and is primarily a matter of individual choice and responsibility, rather than a public health concern. It is crucial to recognize the disproportionate impact of this norm-setting on vulnerable groups such as children and young adults.

### 2.3 Commercial strategies shaping oral health

The range of market, business, and corporate political strategies used by commercial entities, from multi- and trans-national corporations to national oligarchs, to create, sustain, and expand the markets related to oral health–related goods and services, remains largely under-examined in India.

Market strategies create and sustain demand for oral health–related commodities through marketing and pricing practices. Tobacco companies use sponsorships and sports branding to influence young minds, despite regulatory restrictions (24). Across these products, commercial messaging frequently appropriates the language of health (“low calorie”, “natural”) while promoting ideals of hygiene (“fresh breath”), desirability (“white teeth”), and social mobility. Additionally, through targeted pricing and retail penetration, low-income sections are captured as markets for harmful commodities such as ultra-processed foods and cheaper variants of tobacco, alcohol, or sugar-sweetened beverages (25).

Business practices shape the affordability and market viability of oral health-related commodities through taxation, supply and value chain arrangements, and legal and regulatory compliance. Tobacco companies are known for their aggressive tax evasion strategies, and prominent tobacco corporations own several companies in tax havens (26). The recent reduction in Goods and Services Tax (GST) on beedis has been criticized for promoting cheap access to harmful products and reinforcing exploitative labour arrangements in the informal beedi sector. While sugar-sweetened beverages are taxed at higher rates, GST on raw sugar and sugary products has been reduced, enabling widespread consumption of such products with well-established oral health risks. Despite statutory bans and licensing requirements, commodities such as smokeless tobacco and alcohol circulate through small vendors, retail outlets, and brand extensions, particularly in low-income urban and rural settings. Their local production, informal retail, and fragmented distribution make regulation difficult (27). Evidence from tobacco and alcohol regulation points to uneven enforcement of existing laws, product bans, and provisions such as Article 5.3 of the WHO Framework Convention on Tobacco Control (FCTC), which seeks to protect public health policymaking from industry interference (23).

Corporate political strategies include corporate social responsibility initiatives, lobbying, and other forms of engagement by commercial actors with policy and regulatory processes that may shape the governance context in their favor. In India, direct evidence on such strategies in relation to oral health remains limited, although insights from tobacco and alcohol industries suggest their significance. For instance, studies of tobacco control in India have documented industry lobbying through engagement with policymakers, sponsorship of events, mobilization of interest groups, and legal challenges to regulatory measures, illustrating how commercial actors can shape health governance and policy implementation (28). Practices such as CSR activities and sponsorships of cultural and sporting events have enabled commercial actors to maintain public visibility, while lobbying and policy engagement have been documented in relation to taxation, advertising regulation, and enforcement (29).

## 3 Future research directions

This perspective piece situates oral health in India within the framework of commercial determinants of health, analyzing the interlinked pathways through which commercial forces shape both disease burden and access to care. Structural factors, including economic liberalization and weak regulatory environments, define the broader context of these dynamics. Within this context, commercial actors deploy a wide range of strategies to create and sustain markets for oral health-related goods and services. Differential exposure to, and access to, these products, shaped largely by the ability to pay, contribute to widening oral health inequities. These dynamics extend beyond harmful commodities such as tobacco and alcohol to include beneficial goods and services, including dentifrices and oral healthcare.

Currently, oral health research in India shows limited engagement with these upstream commercial and policy determinants and their implications for oral health equity. Understanding these linkages necessitates incorporation of social science and political economy perspectives within oral health research. A predominantly biomedical paradigm is insufficient to examine how commercial and financial actors shape health governance, policy, and regulation. Such a reorientation is imperative, considering the need for several pressing research enquiries.

Key research questions warrant attention to advance oral health research. The extent to which regulatory policies such as smokeless tobacco bans and advertising restrictions are implemented across contexts requires systematic assessment. The continued marketing of harmful commodities despite regulations warrants a serious inquiry. It is crucial to investigate how pricing strategies and retail availability influence the consumption of tobacco, sugar-sweetened beverages, and oral care products, particularly among low-income populations. It is equally important to understand how oral care products are marketed to shape public perceptions of oral hygiene and prevention. One of the most critical enquiries is how state market dynamics, including corporate political activities, influence the expansion and regulation of dental education, services, and oral health-related commodities.

Addressing these questions requires a systems approach that examines the interactions between commercial actors, markets, governance arrangements, health systems, and social determinants of health. Such inquiries will necessitate multidisciplinary research drawing on public health, economics, sociology, political science, and health policy. Approaches such as policy analysis, market analysis, supply-chain analysis, and theory-driven qualitative and mixed-methods research can help elucidate the complex pathways through which commercial forces shape oral health. Combined with the traditional strengths of oral health research in epidemiology and clinical sciences, these approaches can strengthen oral health scholarship and inform policy responses beyond individual behaviour change.

## 4 Conclusion

Using a commercial determinants lens to examine oral health draws attention to the structural and systemic forces shaping both disease burden and access to care in India. This perspective advances a conceptual framework that brings together commodities, structural conditions, and commercial strategies to better understand how these forces interact to produce oral health inequities. It thus shifts the analytical focus beyond individual-level explanations toward the broader political and economic determinants of oral health. These dynamics also raise important questions of equity and social justice, particularly when access to essential oral health goods and services is determined by market forces rather than health needs. Advancing research, regulatory mechanisms, and policy engagement through this lens holds potential to promote more equitable and prevention-oriented oral health systems.

## Data Availability

The original contributions presented in the study are included in the article/Supplementary Material, further inquiries can be directed to the corresponding author.
